# The effect of Telehealth on disease-specific quality of life in patients with heart failure: the Whole Systems Demonstrator Telehealth Questionnaire Study

**Published:** 2012-06-15

**Authors:** Martin Cartwright, Shashivadan Hirani, Lorna Rixon, Michelle Beynon, Helen Doll, Stanton Newman

**Affiliations:** School of Health Sciences, City University, UK; School of Health Sciences, City University, UK; School of Health Sciences, City University, UK; School of Health Sciences, City University, UK; University of East Anglia, UK; School of Health Sciences, City University, UK

**Keywords:** telehealth, heart failure, quality of life, Whole System Demonstrator

## Abstract

**Introduction:**

Primary studies and systematic reviews that have examined the effect of Telehealth (TH) on Health-Related Quality of Life (HRQoL) typically conclude that TH leads to quality of life improvements. The evidence base on which such conclusions rest is characterised by methodologically weak studies that generate equivocal findings. The effectiveness of TH, in terms of quality of life benefits, has yet to be substantiated in high-quality trials.

**Aims:**

Using data from the WSD Telehealth Questionnaire Study we assessed the impact of TH on disease-specific HRQoL, generic HRQoL and psychological outcomes (anxiety and depression) in patients with heart failure, over a 12-month period.

**Design:**

The WSD Telehealth Questionnaire Study is pragmatic cluster-randomised controlled trial evaluating a broad range of patient-reported outcome measures. Participants were recruited from three Sites in the UK (Cornwall, Kent and Newham). The current analyses focus on participants with heart failure.

**Methods:**

Over 500 participants with heart failure completed measures of disease-specific HRQoL (MLHFQ), generic HRQoL (UK SF-12; EQ5D), anxiety (Brief STAI) and depression (CESD-10) at baseline. Follow-up assessments were conducted at 4 and 12 months.

**Results:**

Primary and sensitivity analyses will be presented for the heart failure cohort and interpreted in light of existing WSD findings that examine generic HRQoL in a pooled clinical sample comprising participants with heart failure, COPD and diabetes. Specific findings from the WSD Telehealth Questionnaire Study are embargoed until these analyses have been peer-reviewed and accepted for publication.

**Conclusions:**

Conclusions cannot be released until the analyses have been peer-reviewed and accepted for publication.

